# Properties of artificial neurons that report lightness based on accumulated experience with luminance

**DOI:** 10.3389/fncom.2014.00134

**Published:** 2014-11-03

**Authors:** Yaniv Morgenstern, Dhara V. Rukmini, Brian B. Monson, Dale Purves

**Affiliations:** ^1^Neuroscience and Behavioral Disorders Program, Duke-NUS Graduate Medical SchoolSingapore, Singapore; ^2^Department of Neurobiology, Duke University Medical CenterDurham, NC, USA; ^3^Duke Institute for Brain Sciences, Duke UniversityDurham, NC, USA

**Keywords:** vision, lightness perception, receptive field, efficient coding, empirical ranking, gain control, image statistics, inverse problem

## Abstract

The responses of visual neurons in experimental animals have been extensively characterized. To ask whether these responses are consistent with a wholly empirical concept of visual perception, we optimized simple neural networks that responded according to the cumulative frequency of occurrence of local luminance patterns in retinal images. Based on this estimation of accumulated experience, the neuron responses showed classical center-surround receptive fields, luminance gain control and contrast gain control, the key properties of early level visual neurons determined in animal experiments. These results imply that a major purpose of pre-cortical neuronal circuitry is to contend with the inherently uncertain significance of luminance values in natural stimuli.

## Introduction

Although vision in humans and other mammals is mediated by photons that are emitted or reflected by objects in the environment, to be biologically successful these photons must be related back to their origins by the image-forming apparatus of the eye. Images, however, cannot specify the physical parameters of the objects and conditions in the environment in which behaviors must be executed. The reason is that illumination, surface reflectance, atmospheric transmittance, object size, distance, orientation, and a host of other factors are conflated in images, and cannot be disentangled to indicate these measures of reality (Purves and Lotto, 2011; Purves et al., 2011, 2014). This confound is referred to as the “inverse optics problem.” Since we and other agents rely on vision to succeed in the physical world, images clearly provide *something* that promotes well-adapted perceptions and behaviors; but if images cannot convey these measurable properties of the environment, then what information does vision rely on?

Most approaches to vision have sought to answer this question by characterizing neural response properties in experimental animals (Hartline, 1938; Barlow, 1953; Kuffler, 1953; Hubel and Wiesel, 2005). Studies carried out more than 50 years ago in cat retinal and lateral geniculate neurons (Kuffler, 1953; Hubel and Wiesel, 1961) showed that early level visual neurons have roughly circular receptive fields that comprise a central region that is either excited or inhibited by a small spot of light and a surrounding annular region with opposing polarity. More recent studies of lateral geniculate neurons in cats and monkeys have added to this description of the “classical” receptive field by demonstrating that early level neurons have an overlapping, modulatory suppressive field that gives rise to contrast gain control (Levick et al., 1972; Solomon et al., 2002; Carandini, 2004; Bonin et al., 2005; Alitto and Usrey, 2008). The rationale for these early level neuronal properties is thought by many to be a means of reducing redundancy between information in natural stimuli and limited neuronal responses (“efficient coding”) (Barlow, 1961). The efficient representation of information has been suggested to explain classical center-surround antagonism (Srinivasan et al., 1982), modulation underlying gain control by divisive normalization (Schwartz and Simoncelli, 2001), and the orientation and spatial frequency tuning of neurons in the primary visual cortex (Olshausen and Field, 1996; Bell and Sejnowski, 1997; Rao and Ballard, 1999). Missing, however, is how these responses could lead to successful behavior in the face of the inverse optics problem.

There is a way to contend with the inverse optics problem, although it cannot be solved in a mathematical sense since the needed parameters are not available. Imagine a population of primitive organisms whose behavior is dictated by rudimentary collections of photoreceptors and associated neural connections. As stipulated by Darwinian theory, the organization of both the receptors and their connections in the population is subject to small, random variations in structure and function. By the same token, the photons falling on photoreceptors at any moment trigger responses that vary as well. Accordingly natural selection will tend to instantiate the variations of pre-neural and neural configurations underlying perceptions and behaviors that promote reproduction in the population. As this process is repeated down the generations, increasingly sophisticated vision can emerge, eventually leading to modern visual systems.

**Figure 1:**
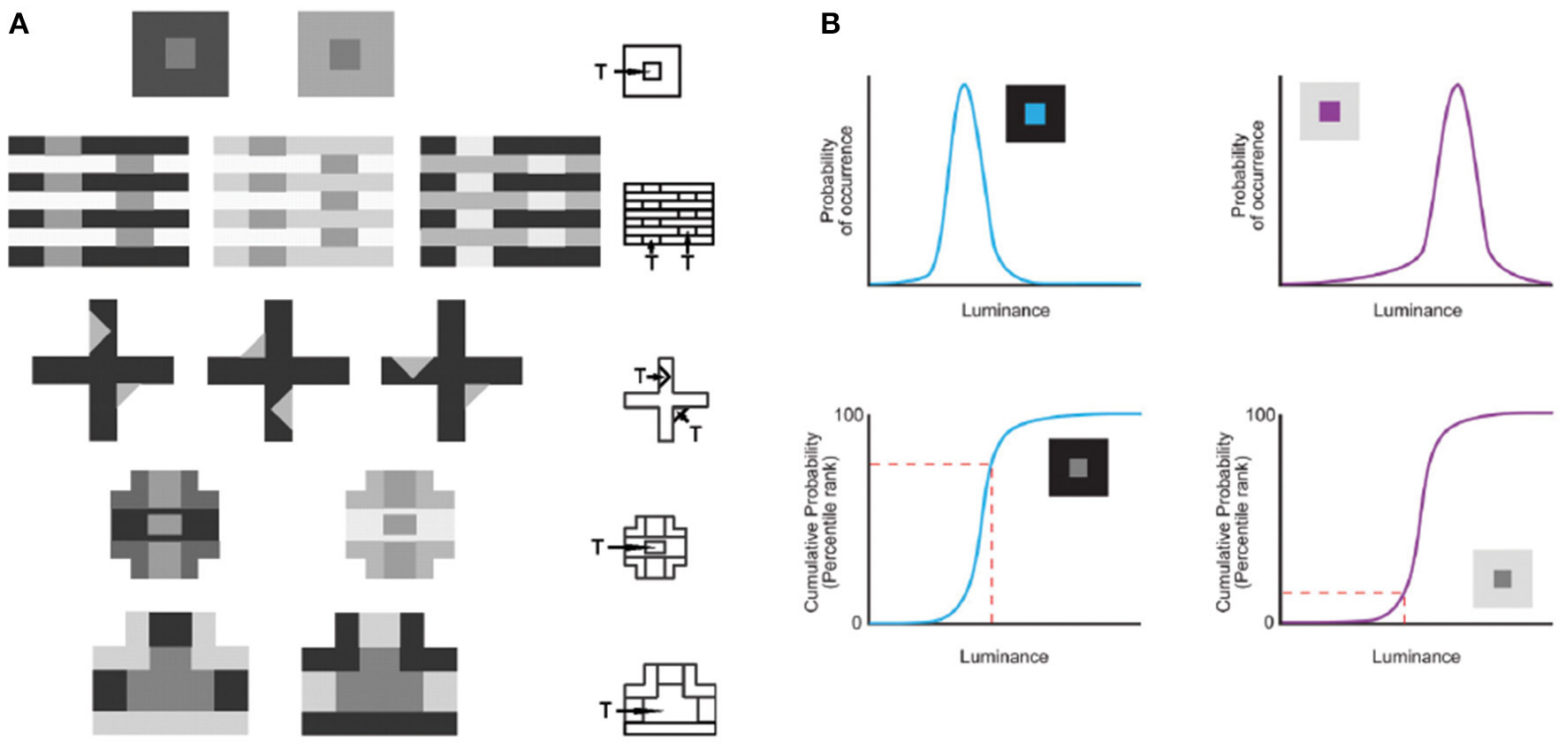
**Predicting lightness percepts using conditional cumulative probabilities. (A)** The same target (“T”) luminance value in different contexts elicits different lightness percepts. The contexts in **(A)** demonstrate the following classical examples (beginning with the top row): simultaneous contrast phenomena, White's effect, the Wertheimer–Benary effect, the intertwined-cross stimulus, and the inverted-T stimulus. **(B)** The lightness perception of the target (“T”) in **(A)** is predicted by the cumulative probability of target luminance value conditioned on the surrounding context luminance values in natural images. As an example we show how this works for the simultaneous contrast stimulus. The probability (top panels) and cumulative probability (bottom panels) of a central target conditioned on low (blue curves on the left) and high (purple curves on the right) luminance surrounds sampled in natural images. The conditional cumulative probability of equiluminant target patches (indicated with a dashed red line) is higher in the low luminance than in the high luminance surround. This higher rank on the cumulative probability function is consistent with human lightness perception of the equiluminant patch in the low luminance surround appearing lighter (adapted from Yang and Purves, 2004, Copyright (2004) National Academy of Sciences, U.S.A.).

In this understanding of vision, the information necessary for reproductive success cannot be conveyed by image features as such, since the properties of the physical world are confounded. For example, whereas perceiving lightness values in proportion to measured luminance values would be of little or no biological use, perceiving lightness in proportion to how often luminance values occurred in the past would have maximized reproductive success. Consistent with this idea, Yang and Purves (2004) showed that the conditional cumulative probability of a target luminance intensity given the context of the surrounding intensities from natural images predicts human lightness percepts elicited by a broad range of stimuli (Figure 1A). They showed that the human perception of an equiluminant target in a low luminance surround appears lighter because the conditional cumulative probability of the target is higher than in a high luminance surround (Figure 1B). This concept of vision shows how humans apparently contend with the luminance inverse problem without information about real world causes (i.e., reflectance, illumination, transmittance and many other factors). Additional psychophysical studies over the last decade have shown that accumulated experience, estimated by the cumulative frequency of occurrence of local patterns of light, predicts the way human subjects see lightness and other basic visual qualities (reviewed in Purves et al., 2011, 2014). Using this strategy, evolved visual biology creates stimuli that tie percepts to reproductive success rather than to the inherently inaccessible metrics of the physical world.

The question we asked in the present report is whether visual circuitry established on this basis leads to the early level neuronal properties evident in animal physiology. We thus evolved the responses of artificial neural networks using a genetic algorithm to match the conditional cumulative distribution functions (CDF) of visual inputs, in accord with perceived luminance, the basic quality elicited by natural stimuli. The results show that artificial neurons comparable to early level neurons in experimental animals exhibit a center-surround receptive field with both luminance and contrast gain control. Although optimization on this basis is consistent with efficient coding principles (Laughlin, 1981; Bell and Sejnowski, 1995; McDonnell and Stocks, 2008), these findings imply that a major goal of early level visual neurons is to contend with the inability of retinal image features to specify the physical world in which animals must act.

## Results

### The network

**Figure 2:**
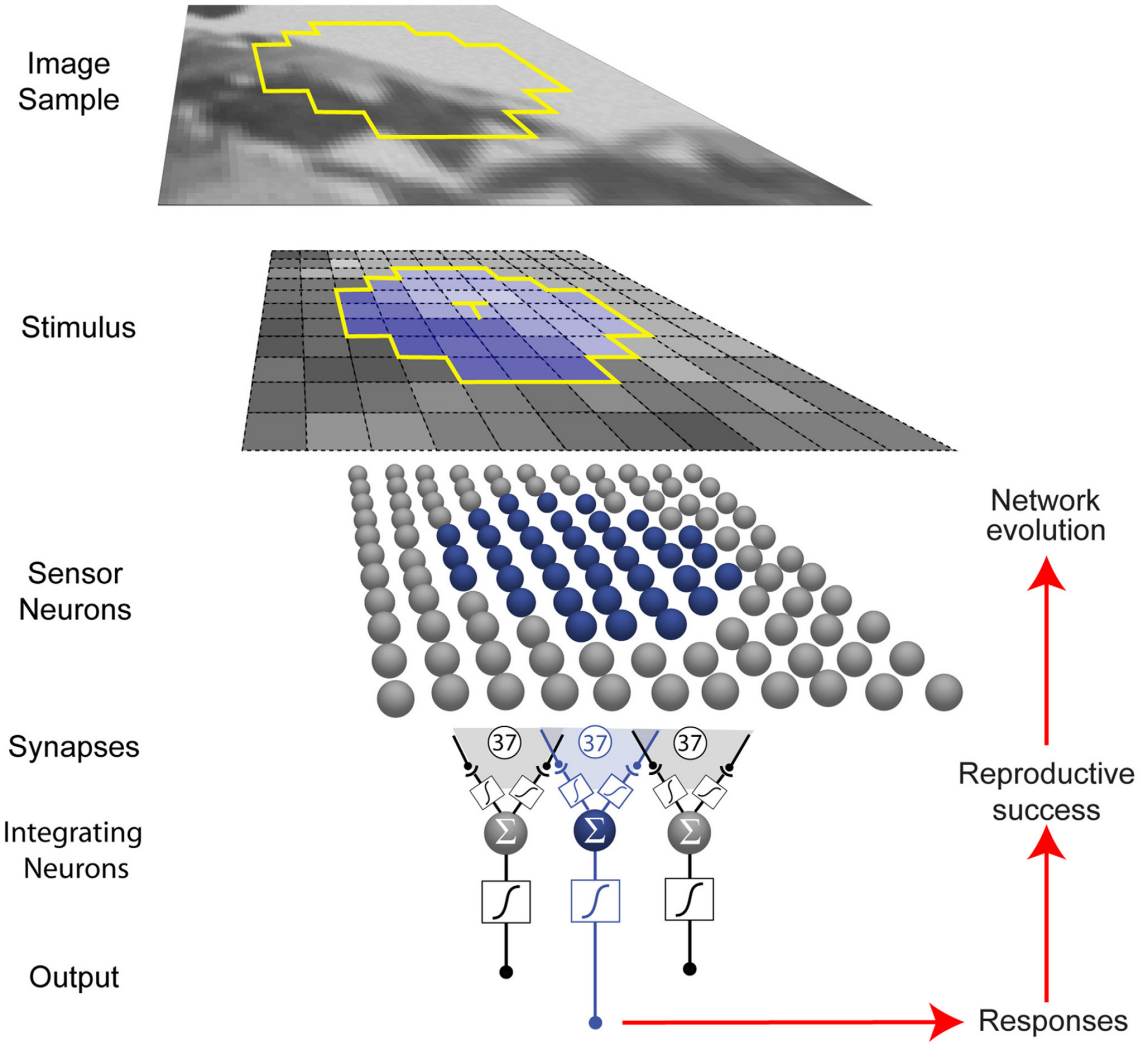
**Network and optimization procedure**. Small patterns of luminance were extracted from natural images (see Materials and Methods). Each network in a population of 500 represented a unit (blue) in a larger array (gray) that received stimuli from a limited region of visual space (yellow outline in the image sample). Neurons are indicated by spheres, and presynaptic endings by black dots. Networks were trained to respond to the luminance value of the central target grid square (T) in the stimulus (blue) with an integrating neuron output value that corresponded to the conditional cumulative probability of the luminance at the central square, given the luminance values of the contextual grid squares.

The networks we used comprised 37 topographically arranged sensor neurons stimulated by local patterns of luminance sampled from a natural image database (Figure 2) (van Hateren and van der Schaaf, 1998). The output of the network was generated from a biologically plausible neuron comprising (1) a nonlinear synaptic transformation of the sensor inputs; (2) an algebraic summation of the transformed inputs; and (3) another nonlinear synaptic transformation at its output (Poirazi et al., 2003). Each network was thus a schematized unit in a larger array that responded to luminance values, the output providing information to what would be the next station in a biological visual system.

In eight independent simulations, the network output values evolved according to how well they matched the conditional CDF of luminance intensities at the central stimulus grid square (the target, T), given the context pattern (C) of the luminance values at the surrounding grid squares (CDF_*T*_(x|C) = *P*(T≤x|C)) in natural images. As described in the Materials and Methods, the conditional CDFs were calculated *a priori* from the image database. Conceptually, these responses rank a target luminance value according to the percentage of luminance values that have occurred more often or less often in the network's experience with patterns of luminance. The networks thus responded to the target value, given the surrounding luminance values.

### Responses

**Figure 3:**
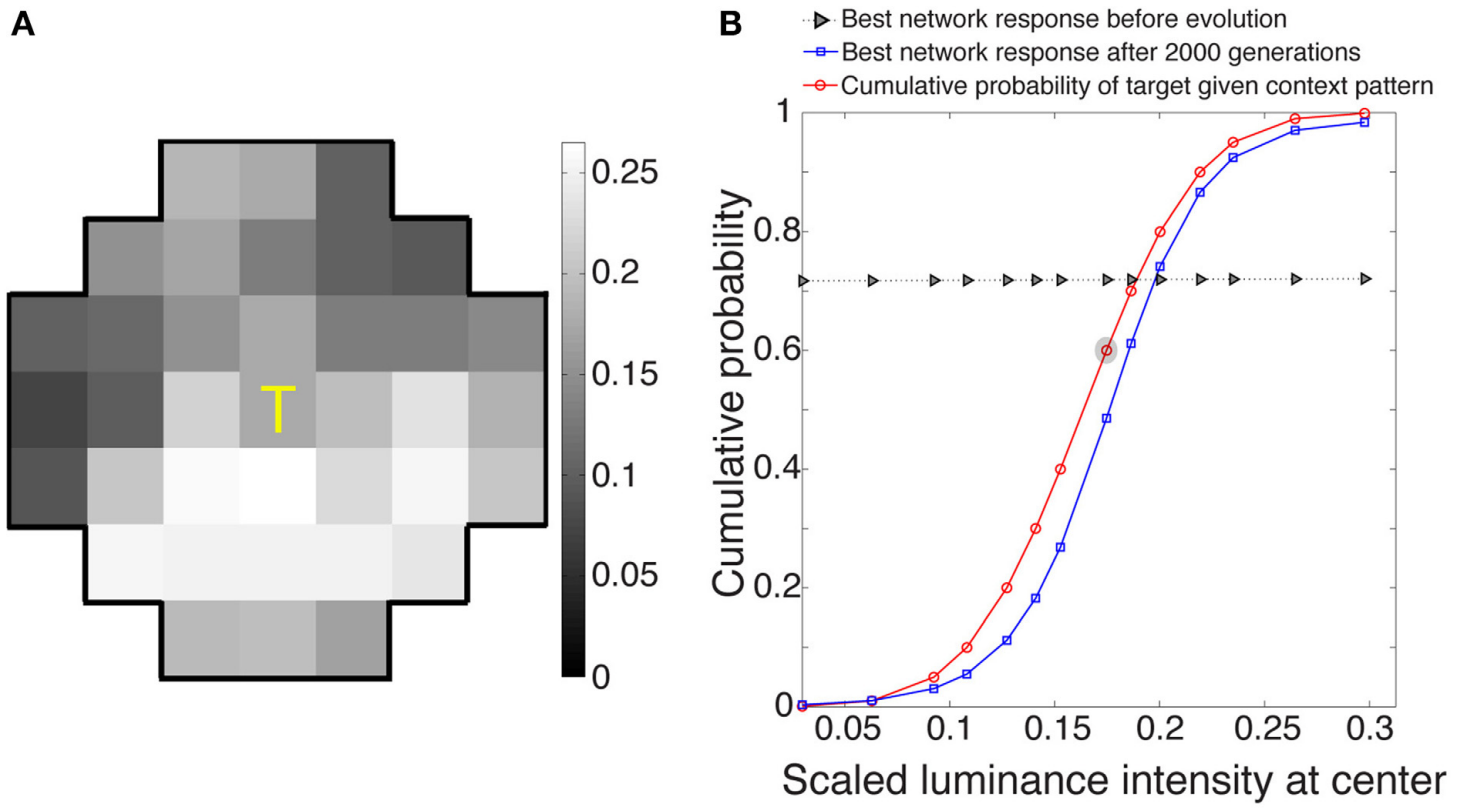
**Network responses to stimuli before and after optimization using a genetic algorithm. (A)** Example of a stimulus drawn at random from the 3500 stimuli presented to each evolving network during its lifetime. **(B)** Evolved network responses (blue) determined by accordance with the conditional CDF of the luminance intensities at the central stimulus grid square, given the luminance intensities of the context pattern in **(A)** (red). The gray triangles show network responses before evolution and blue squares the responses after 2000 generations; the abscissa is the scaled luminance intensity of the central stimulus grid square, and the ordinate is its cumulative probability. As expected from the nature of the paradigm, the evolved responses approximate the cumulative probability of the central grid square given the context grid squares in natural images. The red circle embedded in gray represents the scaled luminance of the central grid square in **(A)**.

The best networks in eight simulations showed output response values that, as expected, approximated the conditional CDF of the central target in a given context (Figure 3). These responses, ranked as percentiles for a given target luminance value, indicate how often central luminance values in that context occurred more frequently than the value of interest and how often less frequently in the cumulative experience of the network's ancestors.

### Response characteristics

#### Classical ON-center/OFF-surround receptive field

**Figure 4:**
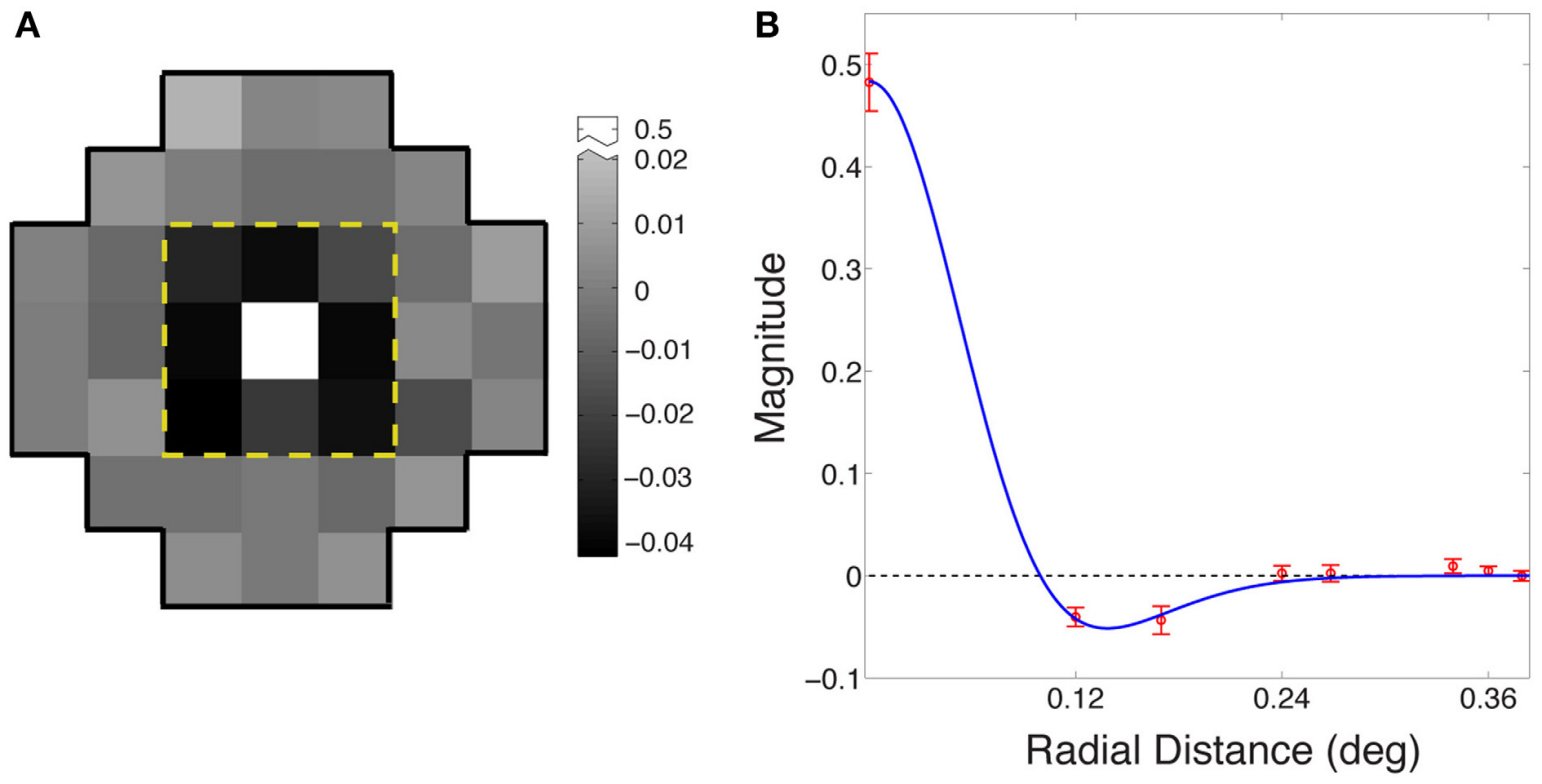
**Evolved receptive field organization of integrating neurons. (A)** The average receptive field for the best performing networks in the eight simulations. The classical ON-center/OFF-surround receptive field in animal visual systems is apparent in the central nine grid squares within the dashed yellow line. The central square is positively valued (arbitrary units), and the eight surrounding squares negatively valued; beyond the yellow line, the input weights of all squares were randomly distributed around zero. **(B)** Radial averages (red) of the receptive field in **(A)**; error bars show ±1 standard errors from the mean. The blue curve is a maximum likelihood fit to a difference of Gaussians function.

Figure 4A shows the average receptive field for the output of the best performing network based on eight simulations (see Materials and Methods). The output response of the integrating neuron to the luminance value of the central grid square in the stimulus increased with increasing luminance, while its response to the immediately surrounding stimulus grid squares decreased as luminance increased. Thus, the integrating neurons ultimately evolved receptive fields that are responsive to the contrast between the central square of a stimulus and the immediately surrounding grid squares.

Figure 4B shows the receptive field of the neurons as radial averages with respect to distance from the center of the receptive field, fit with a difference of Gaussian model RF = *G_center_* − *k_surround_G_surround_* (blue curve), where *G_center_* and *G_surround_* were one-dimensional Gaussian probability densities of widths σ_*center*_ and σ_*surround*_, and *k_surround_* is the relative weight of the surround. The fitted parameters were σ_*center*_ = 0.06° ± 0.001°; σ_*surround*_ = 0.08° ± 0.003°; and *k_surround_* = 0.64 ± 0.01. The ratio of the center to the surround was within the range reported for early level neurons in the cat (Nolt et al., 2004; Bonin et al., 2005).

This center-surround organization is also evident from the pattern of the optimized sigmoids (Supplementary Figure 1). Thus, the sigmoid at the target location showed a positive slope, whereas the sigmoids immediately surrounding the target generally showed a negative slope. The few sigmoids surrounding the target that did not show a negative slope were flat. The receptive field for these neurons was not fully center-surround, indicating that the methods used do not guarantee this receptive field organization (Supplementary Figure 2). We also confirmed that this center-surround organization does not arise when the network responses evolved to match the conditional CDF of white noise patterns (Supplementary Figure 3). In the section below on “Response Properties as a Function of Experience” we also show that the center-surround organization becomes more evident as a function of the network's experience with natural images. These results indicate that experience with natural images, not the network structure, drives the emergence of center-surround receptive fields.

In sum, when responses evolved to match the conditional cumulative probability of targets in naturally occurring surrounds, the integrating neuron in each simulation showed a receptive field organized in much the same center-surround fashion as early level visual neurons in experimental animals. This ON-center/OFF-surround receptive field organization is characteristic of bipolar neurons, retinal ganglion cells, and lateral geniculate neurons (Kuffler, 1953; Hubel and Wiesel, 1961, 1962; Nolt et al., 2004; Bonin et al., 2005).

#### OFF-center/ON-surround neurons

Animal visual systems are also populated by OFF-center/ON-surround neurons that discharge more strongly when the luminance of the receptive field center is less than the surround (DeVries and Baylor, 1997; Chichilnisky and Kalmar, 2002; Ahmad et al., 2003; Balasubramanian and Sterling, 2009). In the present paradigm, OFF-center/ON-surround receptive fields arose when neuronal responses evolved to match the conditional CDF of luminance decrements rather than increments (i.e., the neuronal responses increased when the central luminance intensity decreased).

#### Luminance gain control

**Figure 5:**
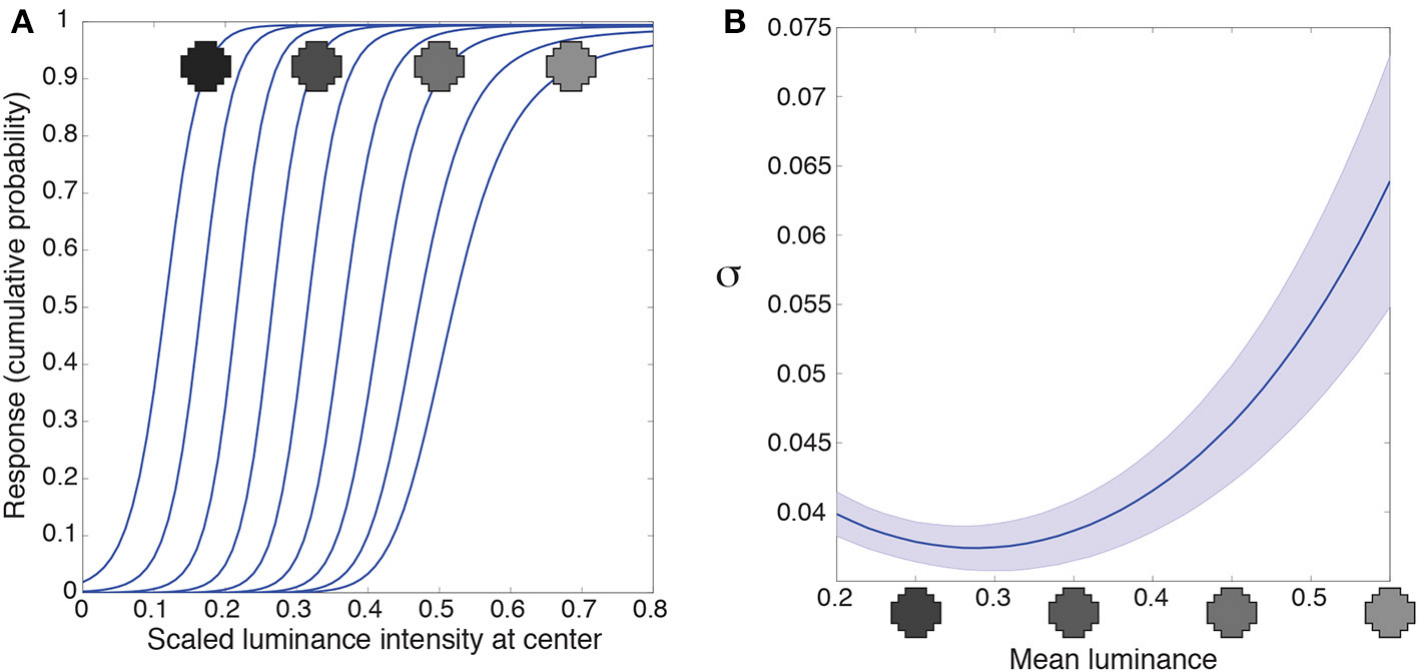
**Luminance gain control. (A)** Responses of a typical integrating neuron to the luminance values of the central grid square as a function of uniform luminance context patterns. The luminance values for the context were within the range the network experienced (mean luminance intensity from 0.2 to 0.55). The neuron's responses to the target vary according to the mean luminance of the surround. **(B)** We quantified the range of input values each neuron responds to for a given surround with the variance (σ) of a cumulative Gaussian fit to its output. The largest variance occurs at high luminance surrounds in **(A)**, meaning that the neurons respond to a broader range of inputs when the surround luminance is high. Blue line shows the mean; shaded region shows ±1 standard errors from the mean. These results accord with the responses of retinal ganglion and lateral geniculate neurons (e.g., Sakmann and Creutzfeldt, 1969).

Early level visual neurons in animals deal with the huge dynamic range of light intensities by luminance gain control (light adaptation), which modulates neuronal responses according to the level of ambient illumination (Sakmann and Creutzfeldt, 1969; Shapley and Enroth-Cugell, 1984; Carandini and Heeger, 2012). In the artificial networks adaptation is an automatic consequence of optimization according to conditional CDFs of natural image patterns. The spatial correlation of luminance values in retinal stimuli drawn from natural images means that a target will always tend to have a lower luminance value in low-luminance contexts, and a higher value in high-luminance contexts. Accordingly, the integrating neuron's response to the luminance in the center of its receptive field is automatically “adapted” to the surrounding luminance values (Figure 5A).

An additional property of luminance gain control in animals is a gain change to intensities in the receptive field center as a function of surround luminance. Retinal ganglion neurons, for example, respond to a broader range of input values as their surround luminance increases (e.g., Sakmann and Creutzfeldt, 1969). We quantified the range of inputs the artificial neuron responds to with the variance, σ, of a cumulative Gaussian fit to the intensity response function from Figure 5A. A larger variance means that the neuron responds to a broader range of inputs. Figure 5B shows the variance (or gain) of the intensity response function is largest in high luminance surrounds. (In Supplementary Figure 4 we discuss an artifact in our methods that leads to the small increase in variance at the lowest luminance surrounds). Thus, networks trained to respond according to the cumulative probability of a target given a surround respond, as do retinal ganglion cells, to a broader range of inputs as a function of the surrounding luminance values.

#### Contrast gain control

**Figure 6:**
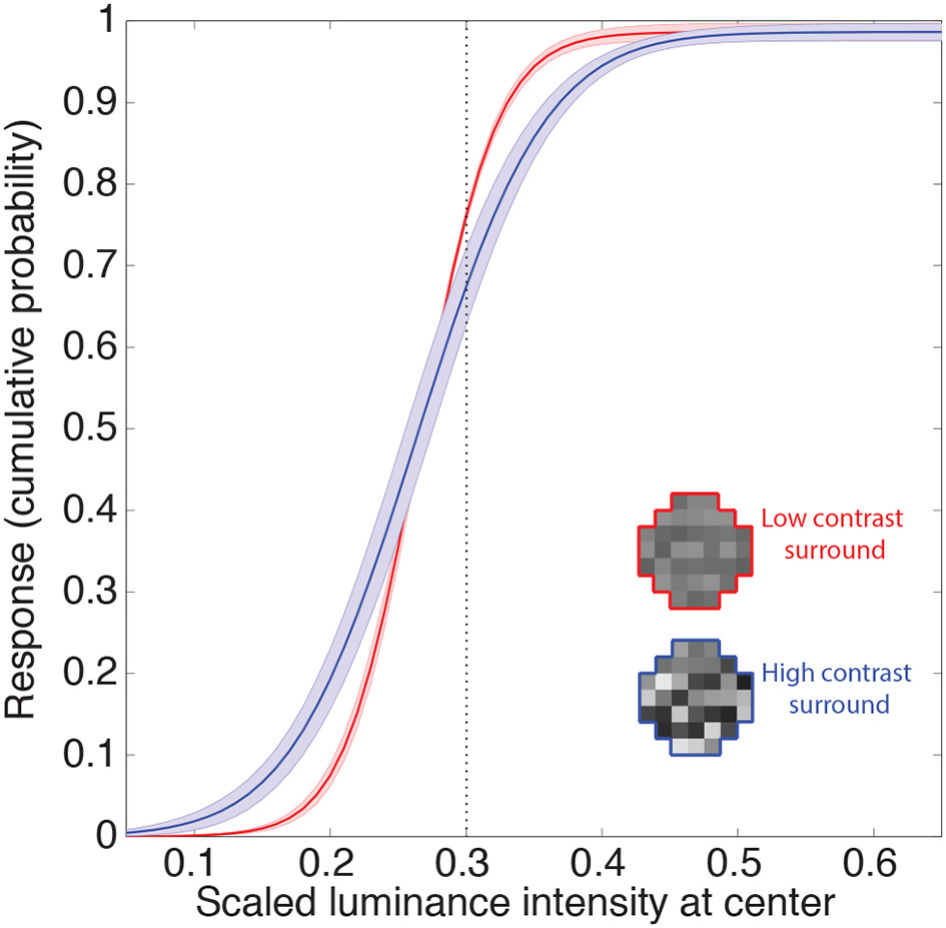
**Contrast gain control**. Example of the integrating neuron's responses to random luminance patterns with either low (red) or high (blue) contrast surrounds. Icons show examples. We recorded the mean network responses to 1000 random luminance patterns with roughly the same mean luminance surrounds (indicated by the dashed line). The solid lines are the means of these responses averaged across all networks. The shaded regions show the 95% confidence intervals. Contrast is defined here as the standard deviation of the random luminance patterns divided by its mean. The contrast of the surround modulates the neuron's response such that the gain is increased at higher contrasts (i.e., an increase in the target luminance results in a smaller increase in the response).

The other gain control mechanism evident in biological neurons entails contrast, and is thought to arise from a suppressive field that overlaps the classical field (see Introduction). This phenomenon is apparent in retinal ganglion cells, and is further enhanced in the lateral geniculate nucleus (Kaplan et al., 1987; Sclar et al., 1990; Carandini, 2004; Bonin et al., 2005). Contrast gain control modulates neuronal responses to input contrast according to average local contrast (Geisler and Albrecht, 1992). Figure 6 shows the responses of the neuron to random luminance patterns with varying contrasts. The contrast of the surround modulates the neuron's input-response function such that at higher contrasts an increase in the target luminance is associated with a smaller increase in response (i.e., the slopes of the input response functions are shallower). Thus, when the target luminance value is higher than the mean of the surround, the neuron's responses will tend to be lower at high contrast. This decrease in response again resembles those in experimental animals (e.g., Geisler and Albrecht, 1992). On the other hand, the neuronal response to a target luminance value that is lower than the mean of the surround becomes larger when embedded in higher contrasts. This increase in response also resembles contrast gain control behavior in biological neurons (e.g., Bonin et al., 2005; Cao et al., 2011). In summary, the output values of the integrating neurons show contrast gain control properties similar to early level visual neurons in experimental animals.

#### Response properties as a function of experience

An evolutionary strategy that can acquire these properties by generalizing based on few samples is more likely to be chosen by natural selection. However, the question of how many types of different patterns need to be experienced to acquire these properties remains. The experiments in Supplementary Figure 5 show the response properties of networks in environments that had limited numbers of patterns, ranging from 70 (5 context patterns presented with 14 target luminance values) to 2800 (200 context patterns presented with 14 target luminance values). The response properties arise after witnessing relatively few patterns, but become clearer as network experience broadens. That few patterns need to be experienced before the network begins acquiring these fundamental properties indicates that this strategy is consistent with biological natural selection.

## Discussion

The present results show that the core properties of early level visual neurons evolve when perception is determined by the conditional cumulative frequency of occurrence of biologically generated stimuli. These observations imply that an important purpose of such neurons is to contend with the inverse problem. In addition to the prediction of basic human psychophysics, this rationale for the characteristics of early level visual neurons is consistent with the observation that fMRI activation in the human lateral geniculate nucleus and primary visual cortex correlates with lightness rather than luminance (Anderson et al., 2009; Boyaci et al., 2010). Success arises not because the physical parameters of the world are recovered but because perceiving lightness in this way aligns perceptions with behaviors that promoted reproductive success, thus contending with the inverse problem as it pertains to luminance (Purves et al., 2014).

### How vision could operate on this basis

How early level biological and artificial neurons evolve this type of connectivity that can initiate apt responses in the absence of information about the physical characteristics of stimulus sources is, in principle, straightforward. Whenever an ancestral agent responded to a stimulus pattern with a percept and ultimately a behavior that was slightly more successful than other members of its cohort, natural selection would have promoted that connectivity in the next generation. Over time, visual system connectivity that led to successful perceptions and behavior would wax in the population, while less useful connections would wane. Although the physical sources of stimulus luminance values are not known, the cumulative probability of luminance patterns determined by experience would in this way successfully inform perception and behavior (Purves and Lotto, 2003, 2011; Yang and Purves, 2004; Purves et al., 2011, 2014). A further consequence of connectivity determined by a fixed set of nonlinear synaptic weighting functions is that its responses to visual input are reflexive. Like any other reflex circuitry, this arrangement has the advantages of assured and rapid responsiveness, while still being able to change based on ongoing evolution and/or activity-dependent neural plasticity during an individual's lifetime.

The gain control properties emerge to estimate lightness of the target on a context-by-context basis. At first glance, the response modulation seems to estimate lightness by discounting illumination. However, the luminance inverse problem is a conflation of many factors, including reflectance, illumination, and atmospheric transmittance. Thus, this modulatory behavior is not estimating lightness by discounting illumination. There are many real-world factors that response properties must contend with and these cannot be disentangled.

### Efficient coding

As noted in the Introduction, a different rationale for why neurons evolve these core properties is efficient coding, which also predicts classical center-surround receptive fields (Srinivasan et al., 1982) and modulatory gain control mechanisms (Schwartz and Simoncelli, 2001). The evolution of a strategy based on accumulated experience that contends with the inverse problem, however, is essential. This is not to say that this strategy is at odds with efficient coding. On the contrary, responses evolved to match the conditional cumulative probability of target luminance values given the luminance values of naturally occurring contexts automatically maximizes a network's information capacity by ensuring that all response levels are used with equal frequency (Laughlin, 1981; Bell and Sejnowski, 1995; McDonnell and Stocks, 2008).

Consistent with efficient coding principles, the network can also be thought of as reducing redundancy in natural images. The conditional CDF approach predicts lightness on a context-by-context basis. By training on the conditional CDF, the network generates values that correspond to lightness rather than brightness via luminance and contrast gain control mechanisms. Since the correlating effects of the illuminant and these other factors cause a major source of redundancy in natural images, the network is decorrelating structure in natural images.

In sum, efficient coding schemes have focused on predicting neural responses without explaining how the visual system resolves the inverse problem. Some investigators have used the conditional probabilities of the filtered outputs of natural images to predict the modulatory behavior of early level neural responses (Schwartz and Simoncelli, 2001). Others have applied efficient coding principles directly on natural images to predict the classical receptive field structure of neurons in the primary visual cortex (e.g., Olshausen and Field, 1996; Bell and Sejnowski, 1997; Rao and Ballard, 1999). The emergence of classical and modulatory early level neuronal properties predicated on perception and successful behavior in the present results implies that a major goal of this circuitry is to deal with *absence* of information about the environment in visual stimuli, rather than its *redundancy*.

### Feed forward network

Other studies have suggested that early level visual stations can signify lightness (Anderson et al., 2009; Boyaci et al., 2010), perhaps even in the LGN (Anderson et al., 2009). Given the extensive higher level feedback to both the LGN and V1, one possibility is that lightness perception is determined by top-down modulation (Boyaci et al., 2007). While the network we used does not address how higher-level modulation might affect lightness perception, it does show that simple feed forward networks can generate outputs that accord with classical aspects of human psychophysics (such as the simultaneous contrast phenomena in Figure 1) in the absence of feedback from the network itself.

Networks with internal feedback may account for lightness percepts in response to more complex patterns. The CDF for White's stimulus, for example, is non-sigmoidal with multiple crossing points (see Figure 3 in Yang and Purves, 2004). Since the present network output necessarily tracks a sigmoid, it cannot respond to the White's pattern in a manner consistent with human perception, whereas a more complex network with feedback connections presumably could.

### Center-surround receptive field organization

The present results thus suggest that the classical center-surround organization apparent in experimental animals arises as a means of generating useful percepts and behaviors despite the fact that visual sensors cannot measure the physical parameters of stimulus sources in the world. The findings also confirm the prediction that a center-surround receptive field organization would emerge if the lateral inhibition evident in smaller networks were extended to larger neuronal arrays (Ng et al., 2013).

### Luminance gain control

Luminance gain control (light adaptation) occurs at most if not all stations of the primary visual pathway, resetting the range of neuronal sensitivity to the ambient luminance so that limited firing rates can convey information about intensities over 10 or more orders of magnitude (Sakmann and Creutzfeldt, 1969). Current theories of gain control propose computational mechanisms that effectively discount the mean luminance of the surround (Srinivasan et al., 1982; Schwartz and Simoncelli, 2001; Carandini and Heeger, 2012). In the present paradigm, however, luminance gain control is a consequence of a nonlinear weighted summation of nearby luminance values (see Supplementary Figures 1, 4), where the weights are determined by accumulated experience of a central target luminance given its surrounding luminance pattern. Thus, like efficiency, luminance gain control emerges automatically as a result of accumulated experience with luminance patterns, without computing the mean luminance of a stimulus or discounting the luminance of the surround (see Figure 5).

### Contrast gain control

The artificial neurons also respond to contrast in a manner that indicates gain control. When a stimulus pattern is characterized by high contrasts, the variance of the target luminance is also high. This effect broadens the conditional CDFs, giving higher than average target luminance value a lower cumulative probability than the same luminance in a low contrast context (see Figure 6), consistent with neurophysiological studies showing lower responses to stimuli at higher contrasts (Geisler and Albrecht, 1992). A further consequence of the broadening of the CDF, however, is that non-preferred stimuli (e.g., OFF-center stimulus presented to an ON-center neuron) embedded in high contrast patterns increase rather than decrease the artificial neurons' responses. This increase to higher contrast patterns is also evident in neurophysiological studies (e.g., Bonin et al., 2005; Cao et al., 2011).

### Limitations

There are several reasons the neural responses in the present study do not exactly match the conditional cumulative probability of a target in natural surrounds (Figure 3). First, the CDFs were based on fits from a normal cumulative function, while the networks were trained to match these curves with a sigmoid. Another reason for some deviation is that the conditional CDFs were computed based on similar patterns, rather than exactly matching patterns. Yet another reason is that a network could have fallen into a local minimum during optimization.

The simple networks we used are limited in several other obvious ways. First, they evolved on the basis of static information, ignoring the dynamic qualities of most visual experience (Benardete et al., 1992; Benardete and Kaplan, 1999). Second, both ON-center and OFF-center receptive fields with opposing surrounds evolve in animal visual systems to provide unambiguous information about luminance increments and decrements (Schiller et al., 1986; Schiller, 1992). Although we separately evolved these two basic types of neurons, the paradigm did not allow them to evolve together. Third, whereas biological neurons show variable responses to identical inputs (Schiller et al., 1976; Dean, 1981; Tolhurst et al., 1983), the responses of the artificial networks to identical inputs were necessarily the same. Finally, the neurons received no feedback from other neurons, a key feature of lateral geniculate neurons (Hubel and Wiesel, 1961). The goal of the paper, however, was not to mimic the properties of biological neurons, but to determine the response properties simple networks needed to resolve the inverse problem presented by retinal luminance.

## Conclusions

Although the paradigm here lacks important features of both biological networks and normal visual experience, artificial neurons evolved to respond to light intensities based on past experience develop both classical and modulatory response characteristics similar to those observed in early level visual neurons. The evolved responses are simple reflexes that, like other reflexes, have the advantage of assured and rapid effects, while still being able to change based on ongoing evolution as well neural plasticity during an individual's lifetime. More importantly, the results show how vision on this basis can successfully address the otherwise daunting fact that the physical characteristics of the world in which we behave are not conveyed by light stimuli.

## Materials and methods

### Networks

All network nonlinearities were modeled as sigmoids with three-free parameters (strength, gain, and horizontal bias) that were randomly initialized near zero (Ng et al., 2013). Thus, there were 114 free parameters; 111 at the first layer and 3 at the second layer.

### Stimuli and their frequencies of occurrence

**Figure 7:**
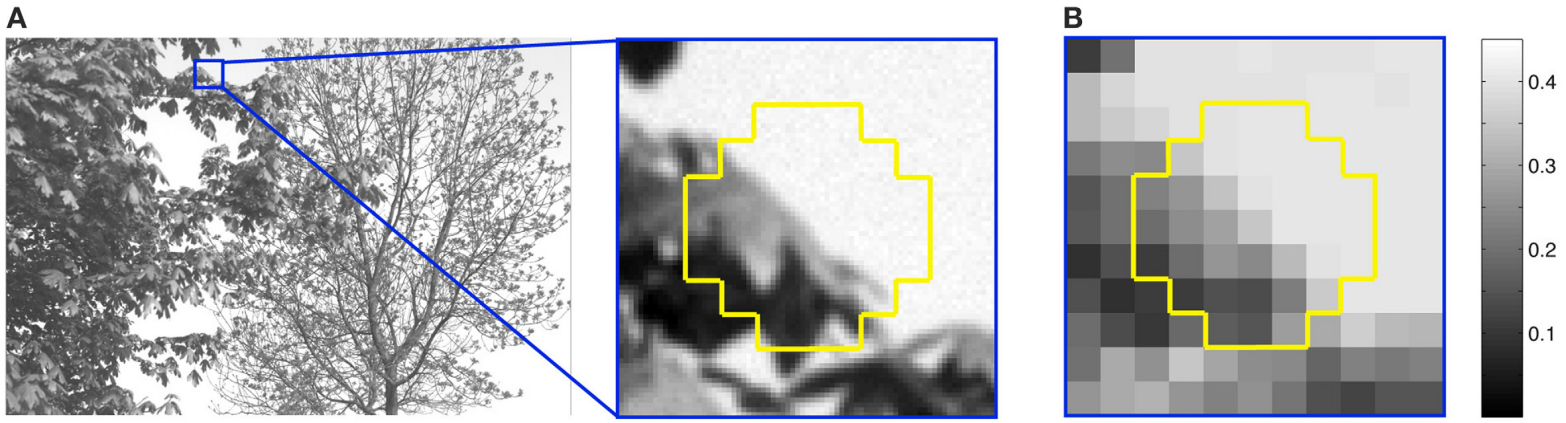
**Extraction of stimuli from natural images. (A)** An image from the database showing the extraction of a typical sample (yellow outline). **(B)** Each sample was divided into 37 grid squares, and the luminance values of 49 image pixels in each square averaged and scaled from 0 to 1 relative to the maximum luminance of 3000 cd/m^2^. For example, the maximum luminance in the sample in **(B)** is 0.45.

The training stimuli presented to the networks were based on three million octagonal samples 0.84° in height and width drawn from 4167 calibrated natural images (Figures 2, 7A) (van Hateren and van der Schaaf, 1998). Only samples with luminance intensities between 0 and 3000 cd/m^2^ were used, since greater intensities occurred too infrequently to provide reliable statistics. The image samples were divided into 37 0.12° × 0.12° grid squares, each of which comprised 49 pixels in the original image; these pixels were averaged and scaled so that the luminance intensities of the 37 stimulus grid squares fell within a 0 to 1 range (Figure 7B). The size of the sample is not critical since natural image statistics are scale invariant over a large range (Ruderman and Bialek, 1994; Huang and Mumford, 1999; Yang and Purves, 2004).

**Figure 8:**
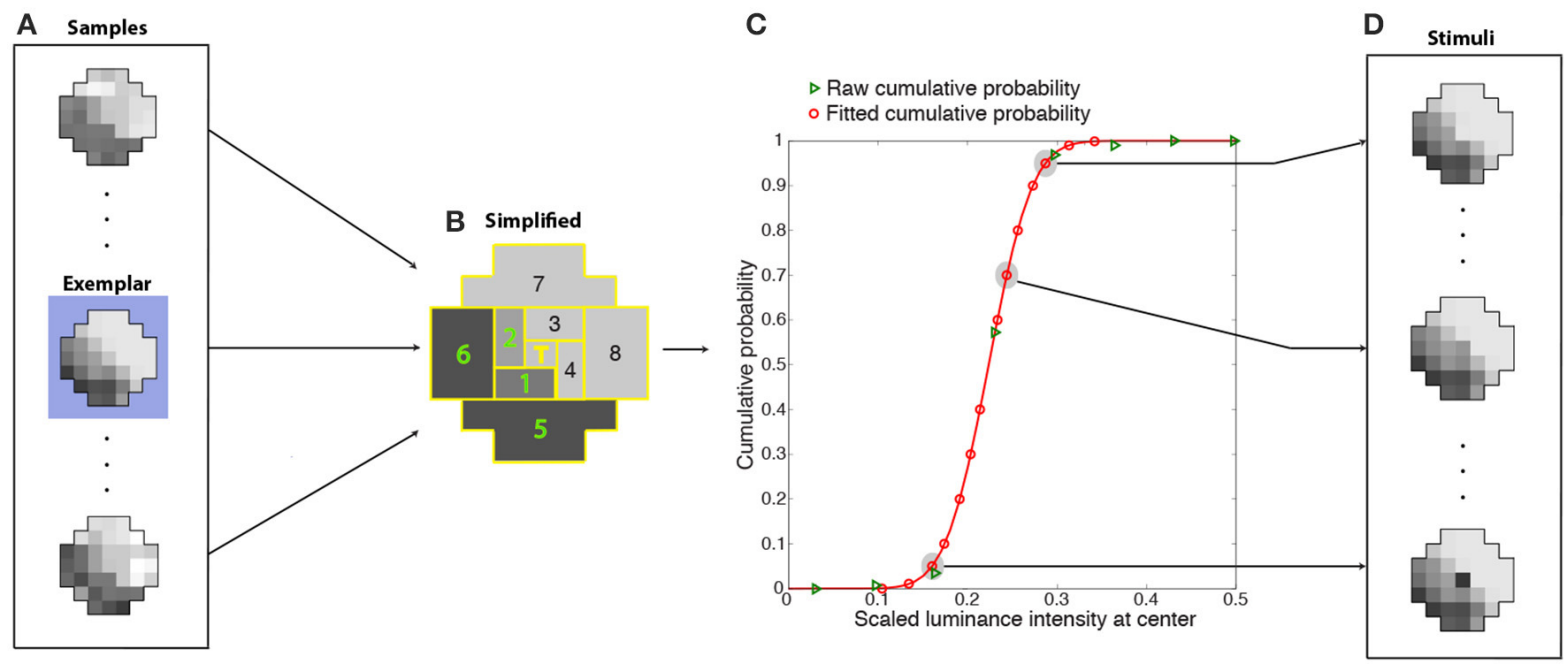
**Determination of cumulative conditional probabilities of the central luminance values in natural stimulus patterns used for training the network**. Luminance patterns **(A)** like the example in Figure 7B were divided into nine regions **(B)** (eight regions tiling around a polar axis plus the central grid square) whose luminance values were further averaged and segregated into 15 bins. The conditional CDFs of the central grid square luminance in these simplified context patterns (green triangles in **C**) were fit with a cumulative Gaussian probability function (red curve). The fit was used to estimate 14 scaled luminance values at the center with cumulative probabilities that spanned a 0 to 1 range (red circles in **C**). During training, the stimuli presented to the networks **(D)** were one of 14 possible luminance values of the central grid square (red circles in **C**) embedded in the context pattern of one of the original (37-grid squares) samples (in this case, the exemplar pattern highlighted in blue in **A**). The ideal response that each network was trained to approximate was the rank on the conditional CDF of the central grid square (T) (see graph).

Given the enormous number of possible patterns of 37 grid squares with many luminance levels (see Figure 7B), we averaged the luminance intensities falling within eight regions tiling a polar coordinate frame around the central target grid square (Figures 8A,B). Binning the (averaged) luminance intensities of these regions into 15 bins reduced the number of unique context pattern surrounding the target from the initial sample size of 3-million (36 grid square patterns with a continuous range of intensities) to 172,547 total patterns (8 averaged regions with 15 discrete intensities), with sufficient recurring patterns to calculate an estimate of the frequency of occurrence of the central grid square luminance values. The rank on the conditional CDF scale of the central grid square luminance in these stimulus patterns thus approximated the rank of central grid square luminance values in generally similar natural scene contexts. In supplementary tests we verified that the optimized neuron's center-surround receptive field is independent of spatial averaging over eight regions (Supplementary Figure 6). For this purpose we trained networks on the basis of the frequency of occurrence 1-D patterns of nine regions (Supplementary Figure 6A). By sampling nine regions rather than 37 and putting the luminance values of each region into 1 of 15 bins, we were able to compute the frequency of occurrence of a target given the eight surrounding grid squares without employing the spatial averaging.

Each training stimulus presented to the networks was one of the original samples (the exemplar in Figure 8A) in one of the more frequently occurring simplified patterns (as described below). The 36-grid square context pattern was chosen as follows. Since many samples (Figure 8A) contributed to each simplified context (Figure 8B), the original sample (the exemplar) had the smallest least-squares deviation from the simplified context pattern. The value at the central grid square for this context pattern was chosen as follows. The conditional CDFs of the central grid square luminance in these simplified context patterns (green triangles in Figure 8C) were fit with a cumulative Gaussian probability function (red curve). The fit was used to recover 14 scaled luminance values at the center with cumulative probabilities that spanned the entire 0 to 1 range (red circles in Figure 8C). Thus, the stimuli presented to the networks (Figure 8D) were one of 14 possible luminance values of the central grid square (the scaled luminance values at the target location corresponding to red circles in Figure 8C) embedded in contextual pattern of one of the original (37-grid squares) samples (Figure 8D). The success of each network in response to a stimulus was determined by how closely its output value matched the conditional CDF of the luminance values of the central grid square, given the luminance values of the context (e.g., Figure 3).

### Simulations

All simulations were carried out in MATLAB using the Genetic Algorithm in the Global Optimization Toolbox. We used a genetic algorithm rather than other optimization routines because it mimics natural selection in the evolution of animal visual systems. Two thousand generations were run for eight different non-overlapping sets of stimuli selected at random (without replacement) from the most frequently occurring context patterns in the database. Each network in a population of 500 was presented with one of the 14 possible central luminance values in each context pattern. Unless otherwise specified (i.e., in the section on “Response Properties as a Function of Experience”), each network was presented with 250 context patterns, thus responding to 3500 different luminance patterns in its lifetime. Using the methods described by Ng et al. (2013), the reproductive rates of networks were determined by how well the output values of the integrating neurons matched the conditional CDF of the central grid square values in Figure 8, thus passing on their connectivity to the next generation. Selection for mating and reproduction was determined by the roulette and random diversification as previously described (Ng et al., 2013).

### Determining the receptive fields of the artificial neurons

**Figure 9:**
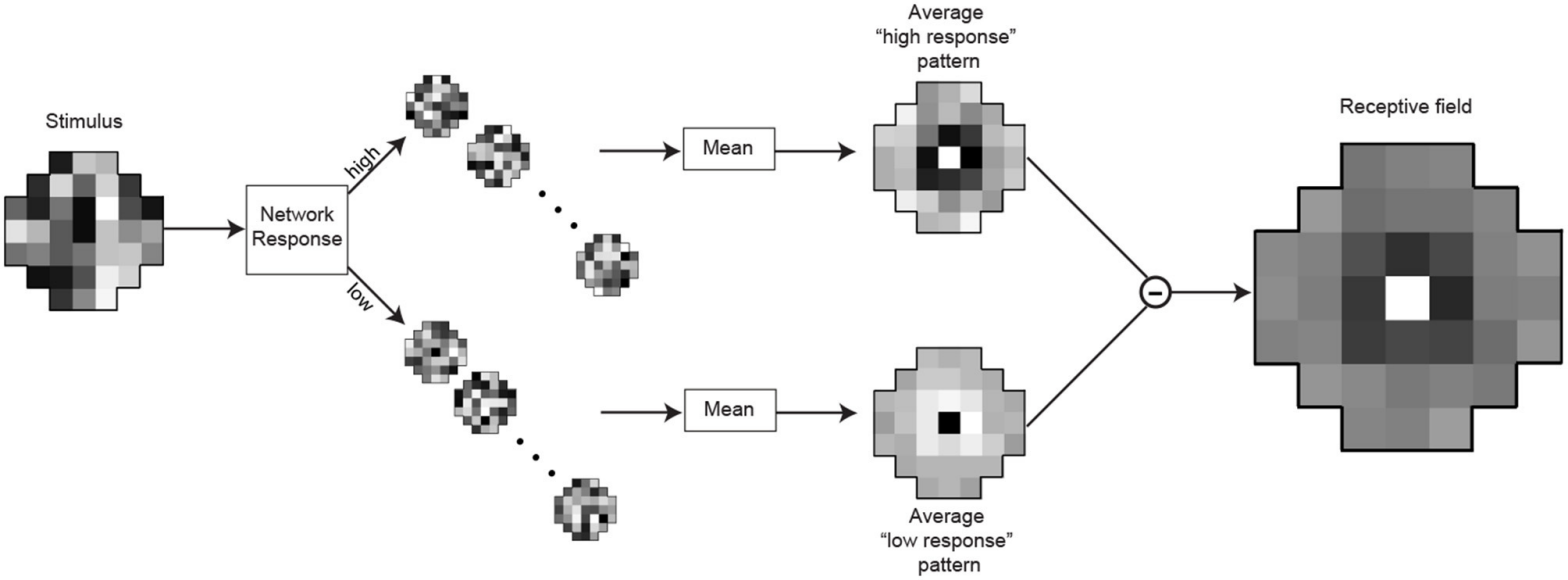
**Determination of the integrating neuron's receptive field**. After training, the responses of the integrating neuron in best network in each simulation were recorded for 15,000 random luminance patterns (example on the left). The stimulus patterns that generated the highest responses (>75% of maximal response) were grouped, as were the lowest responses (<25%). To obtain the receptive field, the mean pattern that generated lowest responses (below) was subtracted from the mean pattern that generated the highest responses (above).

A common technique for the quantitative determination of receptive fields is reverse-correlation (Ringach and Shapley, 2004) or classification image analysis (Murray, 2011). We used an analog of these methods to assess the receptive fields of the optimized neurons (Figures 4, 9). After training the networks to match the conditional CDFs of their input patterns, we recorded the responses to novel random white noise patterns. When a random luminance pattern presented to a fully optimized network elicited a response exceeding a criterion (>75% of the maximum response), we assumed that variations in the stimulus indicated a preferred characteristic of the neuron in question (see Results for details). Lower than average responses (<25% of the maximum response) to random luminance patterns are taken to signify non-preferred stimuli. The receptive field of each integrating neuron was calculated by taking the mean luminance values of the patterns that generated high responses (Figure 9; top) and subtracting the mean luminance patterns that elicited low responses (Figure 9; bottom). A more efficient method for calculating the receptive field would be to incorporate the white noise stimuli with intermediate responses (i.e., between 25 and 75%). When recording from biological neurons the experimenter can collect only a limited amount of data and naturally incorporates all of it to determine the receptive field with a highest possible signal to noise ratio. In contrast, with an artificial network, we could record an unlimited amount of data. Thus, we were able to attain sufficient samples with high responses to calculate a receptive field.

Although we report only the results for the best performing networks in each simulation, networks with errors that ranked as low as 250th out of the population of 500 show similar receptive field characteristics.

## Author contributions

Yaniv Morgenstern and Dhara V. Rukmini were involved in the coding and implementation of the simulations. All authors conceived the experiments and wrote the manuscript.

### Conflict of interest statement

The authors declare that the research was conducted in the absence of any commercial or financial relationships that could be construed as a potential conflict of interest.
