# Supplementary material for: Properties of artificial neurons that report lightness based on accumulated experience with luminance
Source: Front Comput Neurosci. 2014 Nov 3;8:134. doi: 10.3389/fncom.2014.00134 (PMC4217489; doi:10.3389/fncom.2014.00134)
Supplement: Supplementary file 1 [file Image1.PDF]

Supplementary Figure 1

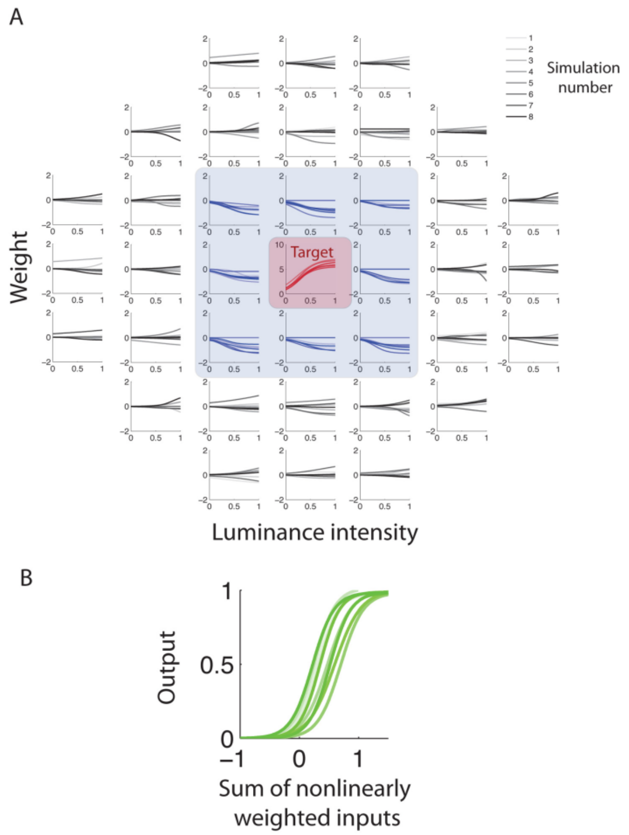

Supplementary Figure 1: The nonlinear transformation functions of the networks after 2000 generations. The transformation functions of the best networks in 8 different simulations (the intensities of the lines in each subplot indicate different simulations). A) The 37 sigmoids that evolved to transform the luminance input. The transfer function at the target location is shown in red; the transfer functions at the immediate surround are shown in blue, and more distant locations in black. The sigmoid slopes at the target were positive, whereas those immediately surrounding the target were generally negative. Note that there was no consistent trend of the transfer functions at more distant loci. B) The nonlinear transformation that converts the sum of the weighted inputs from all the transformations in (A) into the network output response.
