# Supplementary material for: Properties of artificial neurons that report lightness based on accumulated experience with luminance
Source: Front Comput Neurosci. 2014 Nov 3;8:134. doi: 10.3389/fncom.2014.00134 (PMC4217489; doi:10.3389/fncom.2014.00134)
Supplement: Supplementary file 2 [file Image2.PDF]

Supplementary Figure 2

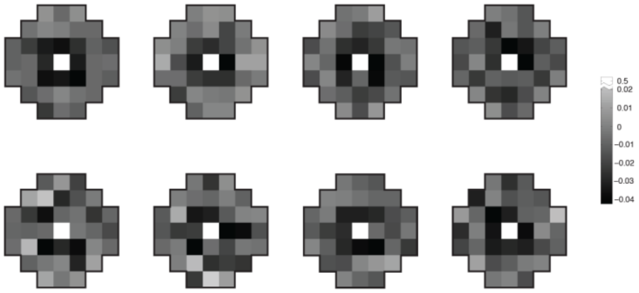

Supplementary Figure 2: Receptive field organization from individual simulations. The receptive field for the best networks in each simulation were not always fully center-surround (e.g., bottom left and top-right).
