# Supplementary material for: Properties of artificial neurons that report lightness based on accumulated experience with luminance
Source: Front Comput Neurosci. 2014 Nov 3;8:134. doi: 10.3389/fncom.2014.00134 (PMC4217489; doi:10.3389/fncom.2014.00134)
Supplement: Supplementary file 3 [file Image3.PDF]

Supplementary Figure 3

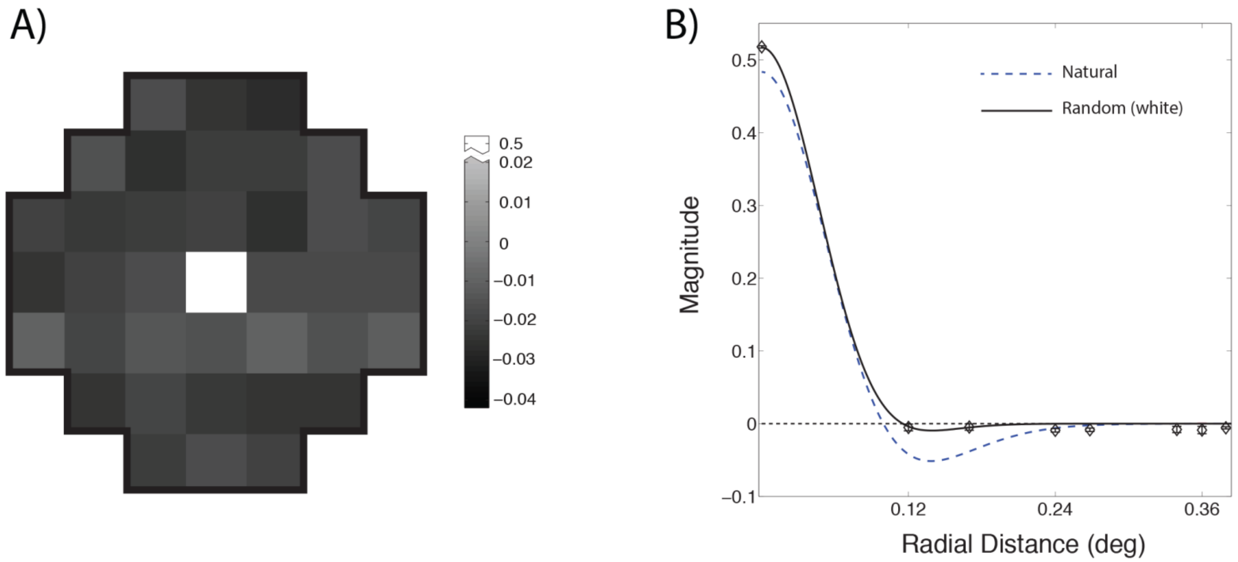

Supplementary Figure 3: Receptive field organization of integrating neuron towards a white noise environment. (A) The average receptive field for the best performing networks in 8 simulations trained according to the conditional cumulative probability of white noise patterns. The patterns from the white noise environment consisted of random Gaussian noise with means ranging from 0.2 to 0.8 and standard deviations of 0.03, 0.04, or 0.05. The outputs to these patterns were based on their associated conditional cumulative Gaussian probability. (B) Radial averages (black) of the receptive field in (A); error bars show ±1 standard errors from the mean. The solid black curve is a maximum likelihood fit to a difference of Gaussians function. The dashed blue curve is the receptive field radial profile derived from networks trained on natural images (re-plotted from Figure 4B). In contrast to the simulations with natural images, the integrating neurons trained on white noise patterns showed no center-surround organization.
