# Supplementary material for: Properties of artificial neurons that report lightness based on accumulated experience with luminance
Source: Front Comput Neurosci. 2014 Nov 3;8:134. doi: 10.3389/fncom.2014.00134 (PMC4217489; doi:10.3389/fncom.2014.00134)
Supplement: Supplementary file 4 [file Image4.PDF]

Supplementary Figure 4

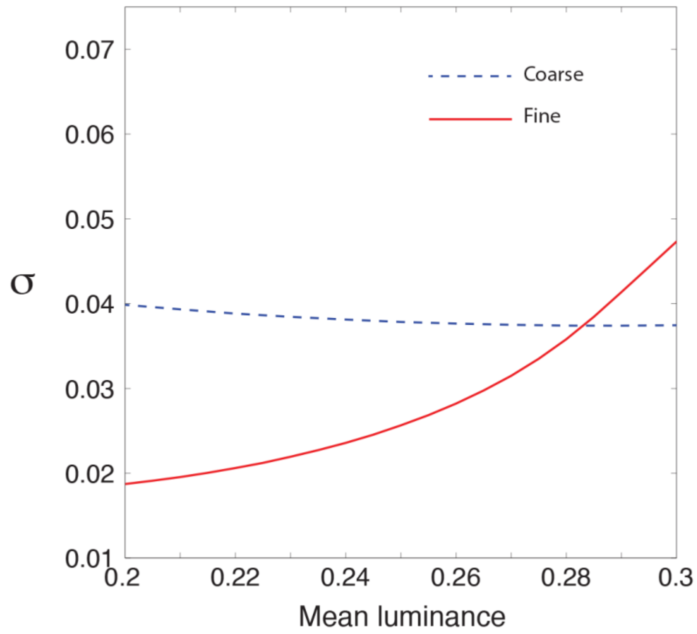

Supplementary Figure 4: Range of input intensities that elicit a response at center location as a function of luminance. The input intensity range that induces a response in retinal ganglion cells (RGC) increases with luminance (e.g., Sakmann and Creutzfeldt, 1969). The variance from a cumulative Gaussian fit to the intensity response functions from Figure 5(A) shows this trend is apparent in the artificial neuron (Figure 5b), except for very low luminance surrounds (dashed blue line). We attribute this inconsistent result to the way we compute the conditional cumulative probability of the target in the surrounding pattern. This bias at low luminance values is a consequence of the equal sized bins we used to compute the conditional cumulative probability of high and low luminance patterns. Since lower luminance patterns have a smaller variance, smaller bin sizes are needed to adequately sample them. This limitation causes the resolution of sampled luminance values to be relatively poor at low luminance, where many patterns are grouped together that would have remained separate if sampled with smaller bin sizes, causing the variance of CDFs to increase. Consistent with this idea, when the bin size over this lower luminance range is made finer (decreased by a factor of two), the network showed smaller gains for the low luminance surrounds (solid red line).
