# Supplementary material for: Properties of artificial neurons that report lightness based on accumulated experience with luminance
Source: Front Comput Neurosci. 2014 Nov 3;8:134. doi: 10.3389/fncom.2014.00134 (PMC4217489; doi:10.3389/fncom.2014.00134)
Supplement: Supplementary file 5 [file Image5.PDF]

Supplementary Figure 5

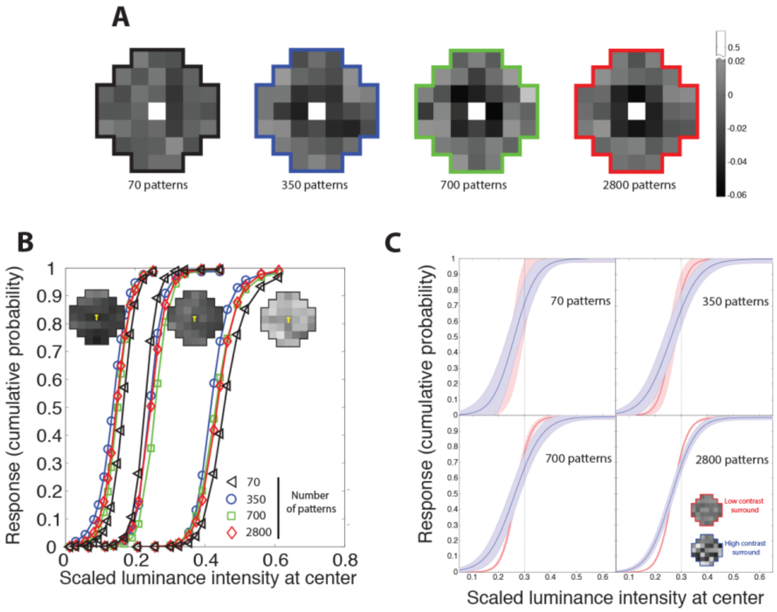

Supplementary Figure 5: Response properties as a function of experience with different patterns. A) The receptive fields of networks in environments with 70 (left), 350 (mid-left), 700 (mid-right), and 2800 (right) different patterns. The center-surround receptive field becomes more visible as a function of experience. Luminance (B) and contrast (C) gain control arises in environments with different number of patterns. Responses are shown for networks in environments with 70 patterns (black triangles), 350 patterns (blue circles), 700 patterns (green squares), and 2800 patterns (red diamonds). Both gain control mechanisms emerge after experience with relatively few patterns.
