# Supplementary material for: Properties of artificial neurons that report lightness based on accumulated experience with luminance
Source: Front Comput Neurosci. 2014 Nov 3;8:134. doi: 10.3389/fncom.2014.00134 (PMC4217489; doi:10.3389/fncom.2014.00134)
Supplement: Supplementary file 6 [file Image6.PDF]

Supplementary Figure 6

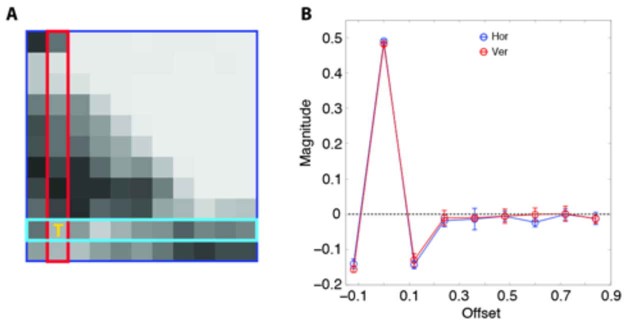

Supplementary Figure 6: Receptive field organization of integrating neurons that experienced a one-dimensional environment. A) A 1-D network trained on environments consisting of 3 million 1-D patterns of 9 samples from natural images extracted either vertically (red) or horizontally (blue). In both cases the networks trained to match the cumulative frequency of occurrence of the target (T) in its surrounding 8 samples. B) The network's 1-D receptive field in both horizontal and vertical environments shows the classical center-surround organization.
